# A variable structure pneumatic soft robot

**DOI:** 10.1038/s41598-020-75346-5

**Published:** 2020-11-02

**Authors:** Wenkai Huang, Junlong Xiao, Zhipeng Xu

**Affiliations:** 1 Center for Research on Leading Technology of Special Equipment, School of Mechanical and Electrical Engineering, Guangzhou University, Guangzhou, 510006 China; 2 School of Mechanical and Electrical Engineering, Guangzhou University, Guangzhou, 510006 China

**Keywords:** Engineering, Mathematics and computing

## Abstract

In this paper, a variable structure pneumatic soft robot is proposed. Its structure is variable in that when it grasps irregular objects, it can adapt to different sizes by active expansion or contraction. Its expansion range is from diameter 200 to 300 mm, its four soft pneumatic actuators (SPAs) can be rotated independently to adapt to different shapes, and it has high flexibility. The active compliant grasping method enables it to capture at the best position, which can improve the success rate of capture and reduce damage to the object being grasped. The experiment proves the effectiveness of the variable structure mechanism, and the proposed soft robot has low cost and a simple manufacturing process, so the mechanism has great application prospects.

## Introduction

The grippers are widely used in industrialization. Since the conventional gripper is generally a rigid mechanism, although it can stably control and grasp very heavy items, its adaptability and safety are not high. To grasp irregular objects and those with different stiffnesses, soft robots show great promise. They are influenced by bionics, soft robots use hyperelastic material, which allows them to passively comply with the object being grasped and interact well with people and have good adaptability^1–3^.

With the rapid development of the field of soft robots, many soft grippers driven by different driving modes have been proposed, such as shape memory alloy actuators (SMAs)^4–8^, electroactive polymer actuators (EAPs)^9–13^, pneumatic grippers^14–16^ or prosthetic jammers^17–19^, self-healing polymers^20^ etc. They all rely on passive compliance for gripping, these actuators either are fixed under the substrate, so under the being grasped same object, the success rate and safety of grasping vary with the shape and size of the object being grasped. At present, most soft grippers have extra mechanisms added to increase gripping flexibility, such as improving grasping ability through variable stiffness^21^. Adding a bending device over the gripper to improve adaptability^22,23^ added a conversion mechanism and a movable sucker, driven by a servomotor; it has four fingers that can be rotated, and there are four grasping modes. Alternatively, two fingers^24^ can be controlled by a ratchet, which can grasp, pinch and envelop objects.

There is still much room for improvement in the existing grippers. For example, in traditional methods, if it is necessary to grasp large objects, a larger soft pneumatic actuator (SPA) length and the distance between them must be designed in advance. In this case, if it is necessary to grasp small objects, the end of SPA is easily affected by the curvature of the object or other external factors, such as dislocation, jitter and so on, which reduces the success rate of grasping small objects, and the appropriate opening size adapted to the size of the object can effectively improve the adhesion and stability of SPA.

In order to meet the requirements of grasping the size and shape of the target actively and passively, and to better apply in the industrial field, a novel adaptive variable structure device is needed to grasp the irregular target stably and safely, which can adapt to the size and shape of the target actively and passively In order to obtain the best grasping strategy. Therefore, this paper proposes a variable-structured pneumatic soft robot. To achieve a simple and compact design and better performance, four crank slider mechanisms are used with five servo motors, and the middle servo motor drives the crank slider. By controlling the rotation of the middle servo motor, the whole mechanism is driven to actively extend and retract to adapt to the size of the object to be grasped. The other four servo motors can rotate independently to adapt to the shape of the object to be grasped. The bending of the SPA realizes the ability of the fingers to bend and grasp.

**Figure 1:**
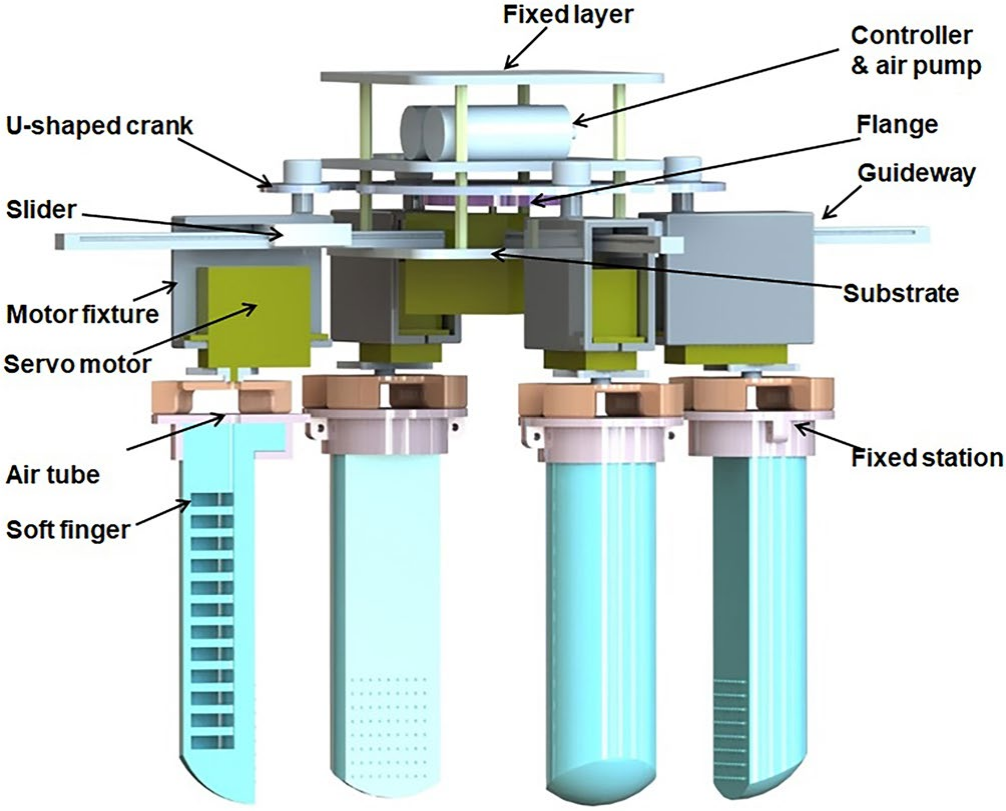
Three-dimensional rendering of soft gripper. In the three-dimensional drawing of SolidWorks, soft fingers are fixed in fixed station, fixed station is fixed in the servo motor which connected with motor fixture. Motor fixture and slider are connected to slide together on the guideway. The servo motor in the middle controls the rotation of U-shaped crank through the flange to drive the opening and closing of four soft fingers. The guideway is fixed in a substrate, and the fixed Layer is used to fix the gripper on the manipulator.

The main contributions of this work are as follows:

A new type of variable structure gripper Fig. 1 is designed and manufactured. It keeps the original continuous bending behavior.

The gripper greatly improves the grasping range of the gripper through five steering gears. It can grasp the target from different sizes, directions and strengths, and has high stability and adaptability for objects of different shapes and sizes.

Kinematic modeling of variable structure, analytical modeling and verification of SPA bending behavior and finite element analysis.

The motion space and grasping strategy of the gripper are analyzed, and the experimental evaluation of the gripper is carried out. The influence of different expansion degrees on the grasping limit weight is compared, which shows the superiority of the variable structure.

## Method

### Design of variable structure platform

**Figure 2:**
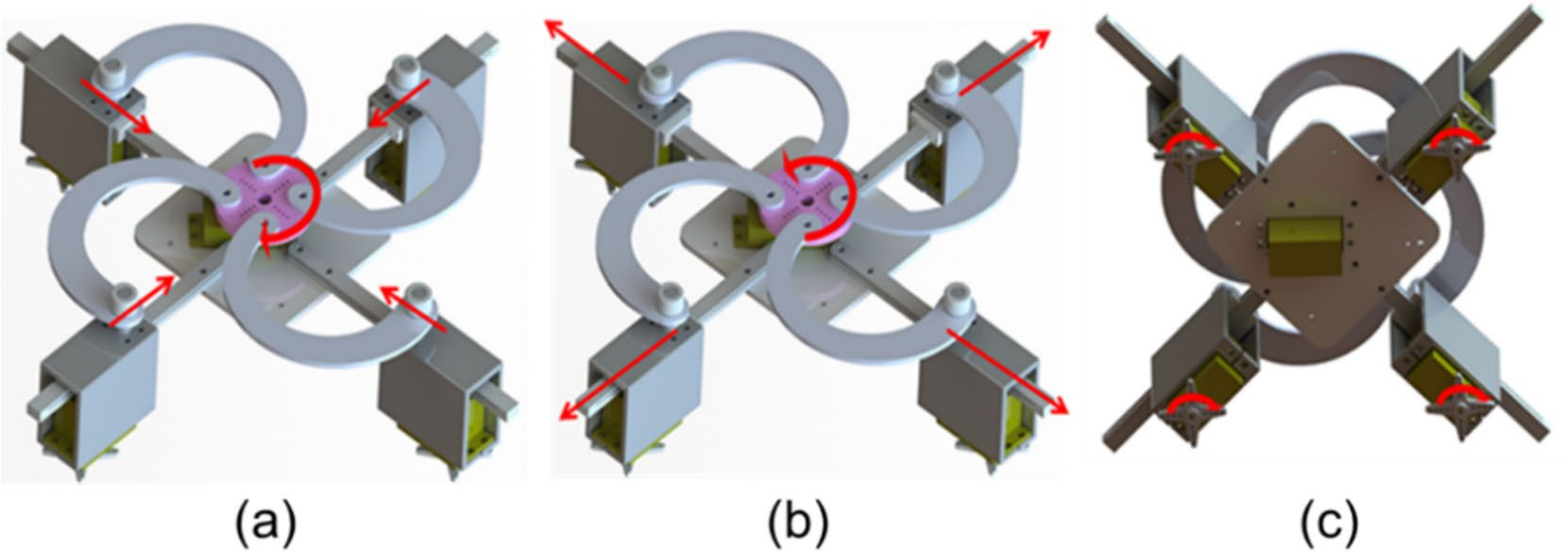
Assembly drawing of the gripper. (**a**) Mechanism contraction movement. (**b**) Mechanism expansion movement. **c** Contraction movement of the body. The rotary arrow represents the direction of rotation of the servomotor, and the straight arrow represents the direction of movement of the component.

In this paper, five independent servomotors and four small air pumps are used as actuators. One of the servomotors is fixed to the substrate and connected to the U-shaped crank through the flange. The purpose is to control the rotation of the middle motor. When the four parts are rotated clockwise, the mechanism is opened, and vice versa. The expansion and contraction range $\mathrm{\varnothing }$max – $\mathrm{\varnothing }$min = 2 * ${R}_{f}$, where ${R}_{f}$ is the radius of the flange. Four independent servomotors are fixed to the motor fixture, the motor fixture is fixed to the slider, and the slider slides on the guideway. The principle and motion of variable structure are shown in Fig. 2.

This mechanism only needs to rotate a servo motor to realize the active expansion and tightening of the whole mechanism, so as to adapt to the size of the object being grasped. Because of the complexity and irregularity of the grasped object in reality, we used four servo motors which were controlled independently to be fixed on the expansion and contraction mechanism and rotated independently to adapt to the shape of the grasped object, allowing the mechanism to grasp in a better direction and position. Four servomotors are used to connect to the SPA. Each SPA is controlled separately, and the rotation of the servo motors drives the rotation of the four SPAs, thereby allowing two kinds of variable structure to be grasped.

### Design and fabrication of SPA

Mold parts of 3D printing PLA + material were used for silicone molding, (a) A layer of release agent (Vaseline) is coated and liquid silicone is injected. (b) Connected to the top mold; (c) Remove the upper mold before the silica gel has been formed but not completely dried, at this stage, the shape of the first layer is made; (d) Connect the middle mold (with the shape of the first layer) and the bottom mold (filled with silicone gel); (e) The mold is removed to complete the production of the actuator model.

**Figure 3:**
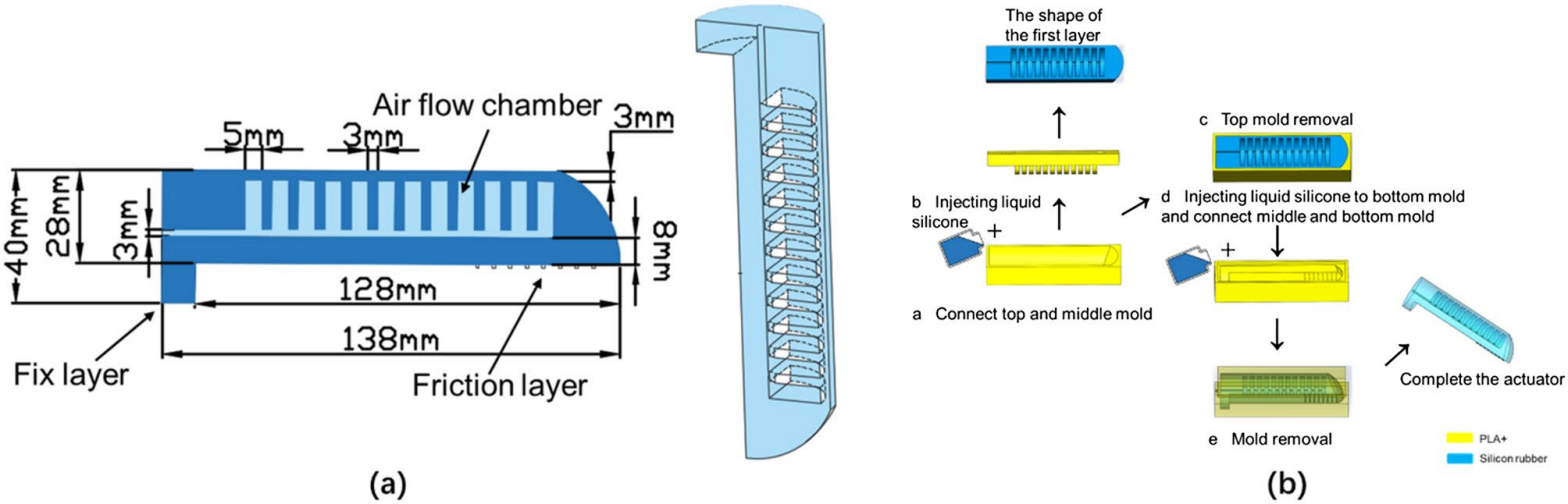
(**a**) The single SPA design; cross-sectional view. (**b**) Manufacturing process of SPA.

As shown in Fig. 3a, there are a total of 12 chambers, and the size of the SPA is 138 mm × 28 mm × 20 mm. The reason for this design is to reduce the energy loss of the radial expansion of the thin wall of the air chamber. This is because the interlayer between the air chambers limits the radial expansion. Although it will reduce the bending capacity, the energy will contribute more to the end moment. We also have a matrix of friction layers (similar to small tentacles) for the SPA that provide passively compliant support^25^, it is made by setting many small cylinder holes on the mold in advance. Small features make it easier to adapt to acquaintance objects and increase the ability to grasp small objects.

We propose a simple method to produce a low-cost SPA, which is made by injecting a three-dimensionally printed mold into a 1:1 A and B mixed 20°medical silicone rubber (PS6600, Shenzhen Yipingyiping Model Material, China). The process is shown in Fig. 3b.

SPAs produced by the above method are simple and can withstand higher actuation pressure than those produced via the adhesive method.

## Result

### Kinematic model of variable structure platform

**Figure 4:**
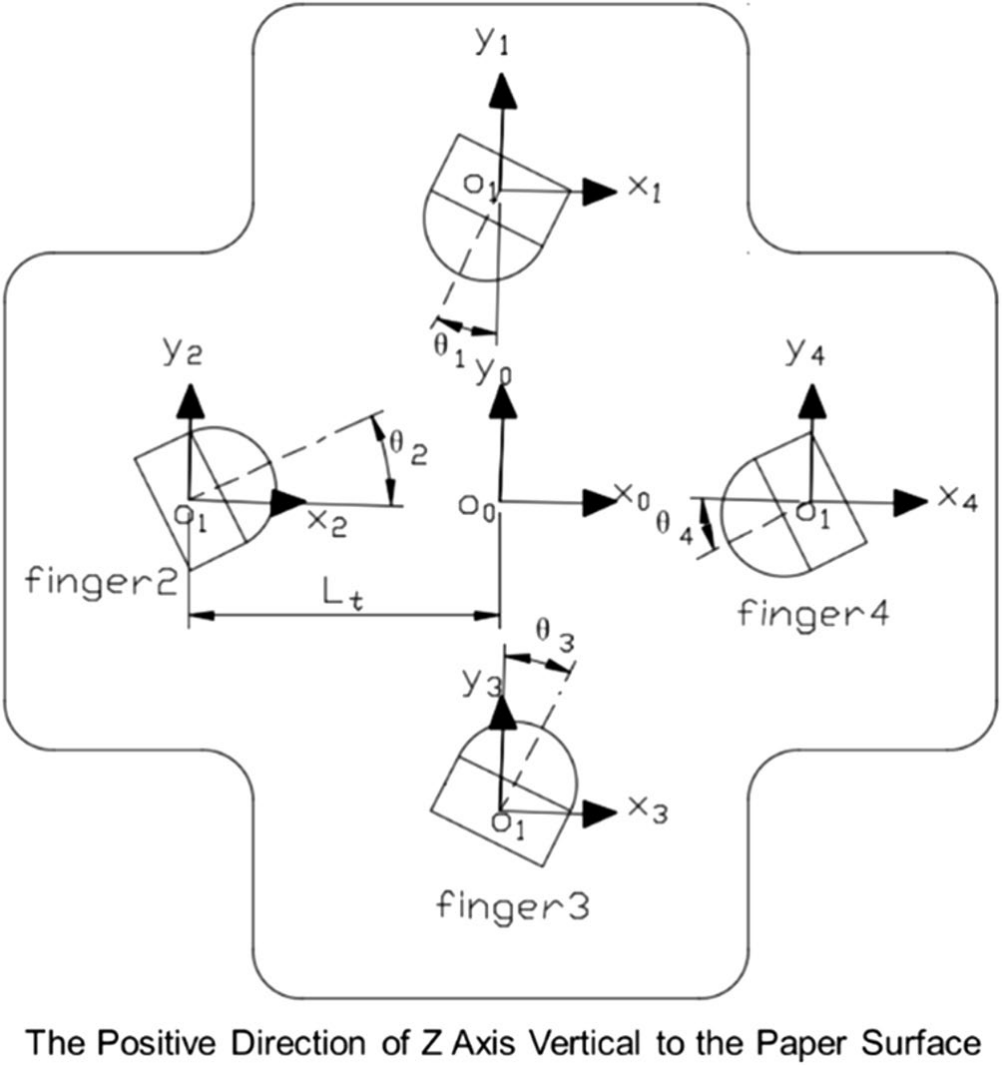
Palm coordinates and fingers coordinates in perpendicular mode (top view).

The palm coordinates and the fingers-root coordinates in the soft gripper are shown in Fig. 4.

1 $$\begin{array}{c}A=\left[\begin{array}{cccc}0& {L}_{0}-{R}_{f}cos{\theta }_{5}& {L}_{SPA0}& 0\\ -({L}_{0}-{R}_{f}cos{\theta }_{5})& 0& {L}_{SPA0}& 0\\ 0& -({L}_{0}-{R}_{f}cos{\theta }_{5})& {L}_{SPA0}& 0\\ {L}_{0}-{R}_{f}cos{\theta }_{5}& 0& {L}_{SPA0}& 0\end{array}\right]\end{array}$$

2 $$\begin{array}{c}B=\left[\begin{array}{cccc}{f}_{x}({P}_{1})sin{\theta }_{1}& {f}_{x}({P}_{1})cos{\theta }_{1}& {f}_{z}\left({P}_{1}\right)& {\theta }_{1}\\ {f}_{x}({P}_{2})cos{\theta }_{2}& {f}_{x}({P}_{1})sin{\theta }_{2}& {f}_{z}\left({P}_{2}\right)& {\theta }_{2}\\ {f}_{x}({P}_{3})sin{\theta }_{3}& {f}_{x}({P}_{1})cos{\theta }_{3}& {f}_{z}\left({P}_{3}\right)& {\theta }_{3}\\ {f}_{x}({P}_{4})cos{\theta }_{4}& {f}_{x}({P}_{1})sin{\theta }_{4}& {f}_{z}\left({P}_{4}\right)& {\theta }_{4}\end{array}\right]\end{array}$$

3 $$\begin{array}{c}C=\left[\begin{array}{cccc}\frac{-sin{\theta }_{1}}{abs(sin{\theta }_{1})}& \frac{-cos{\theta }_{1}}{abs(cos{\theta }_{1})}& -1& 1\\ \frac{cos{\theta }_{2}}{abs(cos{\theta }_{2})}& \frac{-sin{\theta }_{2}}{abs(sin{\theta }_{2})}& -1& 1\\ \frac{sin{\theta }_{3}}{abs(sin{\theta }_{3})}& \frac{cos{\theta }_{3}}{abs(cos{\theta }_{3})}& -1& 1\\ \frac{-cos{\theta }_{4}}{abs(cos{\theta }_{4})}& \frac{sin{\theta }_{4}}{abs(sin{\theta }_{4})}& -1& 1\end{array}\right] \end{array}$$

4 $$\begin{array}{c}D=\left[\begin{array}{cccc}{x}_{1}^{t}& {y}_{1}^{t}& {z}_{1}^{t}& {\theta }_{1}^{t}\\ {x}_{2}^{t}& {y}_{2}^{t}& {z}_{2}^{t}& {\theta }_{2}^{t}\\ {x}_{3}^{t}& {y}_{3}^{t}& {z}_{3}^{t}& {\theta }_{3}^{t}\\ {x}_{4}^{t}& {y}_{4}^{t}& {z}_{4}^{t}& {\theta }_{4}^{t}\end{array}\right]=A+B.*C\end{array}$$

The relevant symbols are explained in Table 1. In this design, the grasping space depends on the flange radius ${\mathbf{R}}_{{\varvec{f}}}$ and the SPA bending degree. We set ${\mathbf{R}}_{{\varvec{f}}}$=25 mm. To meet the design’s grasp space requirements, we designed the SPA dimensions as described above.

**Table 1:** Nomenclature for kinematic model of variable structure platform.

Symbol	Description
${\theta }_{1}\sim {\theta }_{4}$	Servomotor rotation angle for controlling SPA, when the pointer points to the origin, ${\theta }_{1}\sim {\theta }_{4}=0^\circ$, clockwise is positive and counterclockwise is negative
${\theta }_{5}$	The rotation angle of the middle servo motor, when the mechanism is in tightening state, ${\theta }_{5}$=0°, clockwise is positive and counterclockwise is negative
${P}_{1}\sim {P}_{4}$	Input pressure of SPA
${\mathrm{L}}_{0}$	The maximum distance between the center of SPA and the origin when the mechanism is fully opened
${R}_{f}$	Radius of the flange
${L}_{SPAO}$	Initial length of SPA
${f}_{x}\left(P\right)$	Horizontal displacement at the end of SPA
${f}_{z}\left(P\right)$	Vertical displacement at the end of SPA
A	XY plane transformation matrix driven by servomotor 5
B	XYZ space matrix caused by the SPA
C	Transfer matrix generated by servomotors 1 ~ 4
D	Spatial location and direction matrix of the SPAs ends

### Mathematical model of the SPA

Because the stress and strain are practically linear in the low-strain region, for the convenience of calculation, by linear fitting of hyperelastic material in small deformation part, we get elastic modulus E = 250,000 Pa, Poisson’s ratio u = 0.48.

Since the internal chamber of the SPA is complex, it is necessary to simplify the mathematical model of the SPA. To do so, we propose the following assumptions:

The limiting part of the SPA is difficult to elongate and acts as a limiting layer, so it can be approximated as being non-axially stretchable.

The deformation of each chamber inside the SPA is the same, and the bending of the soft jaw is considered continuous, so the total bending angle can be linearly superimposed by the bending deformation of a single cavity.

Since the SPA of the design has a large number of chambers connected in series, the large bending deformation of the SPA is realized, and the deformation of each chamber is not large; thus, within the range of low pressure, it can be considered a small deformation^26^.

**Figure 5:**
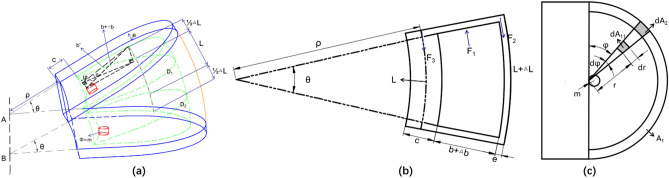
(**a**) Single chamber solid model. The blue line indicates an expansion layer, the red line indicates circular notches, and the green line indicates a cavity. (**b**) Longitudinal section of one layer cavity. ${\rm M}_{1}$ is the moment at which the air pressure acts on the upper end surface of the chamber, ${\rm M}_{2}$ is the moment generated by the pulling force of the thin wall on the side of the chamber, and ${\rm M}_{3}$ is the binding force of the limiting layer. **c** Cross section of one layer cavity.

As shown in Fig. 5a, this is a single chamber solid model. When filling with compressed gas, due to the role of the limiting layer, each cavity will bend at an angle of θ, and the rotation axis is AB axis. ρ is the radius of curvature, C is the thickness of the limiting layer, e is the thickness of the upper and lower end faces, and the red line describes the circular notches of the cavity, with a diameter of M, the blue line describes the upper and lower end faces of the cavity, and the green line describes the internal contour. For the purpose of analysis and calculation, planes D_1_ and D_2_ are parallel to each other and tangent to the interior of the cavity. The distance between the two parallel planes is the original length L, it can be known that the distance from the upper end face to D_1_ is equal to the distance from the lower end to D_2_ is equal to $\frac{1}{2}\Delta\mathrm{L}$.

The upper end surface of a single chamber is analyzed, and under the action of internal air pressure, the upper end surface undergoes elongation deformation, which is obtained by Hooke’s law:

5 $$\begin{array}{c}\Delta b=\frac{{F}_{N}b}{E{A}_{1}}=\frac{\frac{P{S}_{1}}{2}b}{E{A}_{1}}=\frac{Pb\bullet {S}_{1}}{2E\bullet {A}_{1}}=\frac{Pb\bullet \pi bL}{2E\bullet \pi be}=\frac{PbL}{2Ee}\end{array}$$

where P is the internal pressure of the cavity, ${S}_{1}$ is the area of the thin wall on the side of the chamber, and ${A}_{1}$ is the cross-sectional area of the upper end face of the expansion layer. In the case of unfolding into a plane, for the convenience of calculation ${A}_{1}$ can be approximated as a rectangle.

As shown in Fig. 5b, the axes of rotation of the generated moments pass through the axis where the center of the circle is located. Therefore, referring to the method addressed in^27^, the moment balance equation is as follows:

6 $$\begin{array}{c}{\rm M}_{1}={\rm M}_{2}+{\rm M}_{3}\end{array}$$

Analyze the semi-circle of the upper end face, as shown in Fig. 5c.

Take a small area of micro-element:

7 $$\begin{array}{c}d{A}_{11}=rdrd\varphi  \end{array}$$

where $r$ is the integral radius,$\varphi$ is the integral angle.

The pressure exerted by the gas on this small area of micro-element is:

8 $$\begin{array}{c}d{F}_{11}=Pd{A}_{11}=Prdrd\varphi  \end{array}$$

The torque generated by the gas pressure on the area of micro-element is:

9 $$\begin{array}{c}d{\rm M}_{11}=d{F}_{11}{L}_{11} \end{array}$$

where ${L}_{11}$ is the force arm generated by the gas pressure on the area of micro-element:

10 $$\begin{array}{c}{L}_{11}=r\mathrm{sin}\varphi +\frac{c}{2}+\frac{L}{\theta }  \end{array}$$

Then combining (7), (8), (9), (10) the torque ${\rm M}_{11}$ generated by the semi-circular gas pressure on the upper end face can be expressed as:

11 $$\begin{array}{c} {\rm M}_{11}={\int }_{0}^{\pi }{\int }_{0}^{b+\Delta b}\mathrm{Pr}\left(r\mathrm{sin}\varphi +\frac{c}{2}+\frac{L}{\theta }\right)drd\varphi =\frac{2}{3}P{\left(b+\Delta b\right)}^{3}+\frac{1}{2}P\pi \left(\frac{c}{2}+\frac{L}{\theta }\right){\left(b+\Delta b\right)}^{2} \end{array}$$

Since the circular notch on the end face does not produce torque, to calculate the accuracy, the torque of the circular notch on the end face must be subtracted. Similarly, the torque generated by the area of the trachea can be calculated as:

12 $$\begin{array}{c}{\rm M}_{12}={\int }_{0}^{2\pi }{\int }_{0}^{m}d{F}_{12}{L}_{12} ={\int }_{0}^{2\pi }{\int }_{0}^{m}P\eta \left(\eta \mathrm{sin}\beta +m+\frac{c}{2}+\frac{L}{\theta }\right)d\eta d\beta =P\pi {m}^{2}\left(m+\frac{c}{2}+\frac{L}{\theta }\right) \end{array}$$

where $\eta$ is the integral radius of the circular notch on the end face and $\beta$ is the integral angle.

Therefore, the torque generated by the gas pressure on the upper end face is:

13 $$\begin{array}{c}{\rm M}_{1}={\rm M}_{11}-{\rm M}_{12}=\frac{2}{3}P{\left(b+\Delta b\right)}^{3}+\frac{1}{2}P\pi \left(\frac{c}{2}+\frac{L}{\theta }\right){\left(b+\Delta b\right)}^{2}-P\pi {m}^{2}\left(m+\frac{c}{2}+\frac{L}{\theta }\right)\end{array}$$

Next, the thin wall on the side of the chamber is analyzed. Under the action of air pressure, the thin wall of the cavity will elongate. Since the SPA is made up of a plurality of chambers in series, in the case of large bending deformation of the SPA, the thin wall of each chamber will experience a small radial deformation, which is treated as negligible to simplify the calculation. According to the geometric relationship shown in Fig. 5a,b, the approximate bending arc length can be replaced by an $\Delta L$ approximation:

14 $$\begin{array}{c}\Delta L=2{\mathrm{b}}^{\mathrm{^{\prime}}}sin\theta =2\left(b+\Delta b\right)\mathrm{sin}\varphi \mathrm{sin}\theta  \end{array}$$

where ${\mathrm{b}}^{\mathrm{^{\prime}}}$ is the length of the extended upper end face.

Since $\theta$ is small, it is available:

15 $$\begin{array}{c}\mathrm{sin}\theta \approx \theta  \end{array}$$

16 $$\begin{array}{c}\Delta L=2\theta \left(b+\Delta b\right)\mathrm{sin}\varphi  \end{array}$$

Take a small area of micro-element as follows:

17 $$\begin{array}{c}d{A}_{2}=e\left(b+\Delta b\right)d\varphi  \end{array}$$

$dF_{2} = \sigma dA_{2}$During the elongation of the thin wall of the chamber, a tensile force is generated:

18 $$\begin{array}{c}d{F}_{2}=\sigma d{A}_{2} \end{array}$$

19 $$\begin{array}{c}\sigma =\varepsilon E \end{array}$$

20 $$\begin{array}{c}\varepsilon =\frac{\Delta L}{L}  \end{array}$$

where $\sigma$ is the stress,$\varepsilon$ is the strain.

Therefore, $d{F}_{2}$ is expressed by substituting (16)-(20) as

21 $$\begin{array}{c}d{F}_{2}=\frac{2\theta Ee{\left(b+\Delta b\right)}^{2}\mathit{sin}\varphi d\varphi }{L} \end{array}$$

The torque generated by the tension of the area micro-element is:

22 $$\begin{array}{c}d{M}_{2}=d{F}_{2}{L}_{2} \end{array}$$

where ${L}_{2}$ is the force arm of the tensile force of the area of micro-element, which is obtained by the following geometric relationship:

23 $$\begin{array}{c}{L}_{2}=\left(b+\Delta b\right)\mathit{sin}\varphi +\frac{c}{2}+\frac{L}{\theta }  \end{array}$$

Then the moment generated by the pulling force of the thin wall on the side of the cavity is:

24 $$\begin{array}{c}{M}_{2}={\int }_{0}^{\pi }\frac{2\theta Ee{\left(b+\Delta b\right)}^{2}\mathit{sin}\varphi }{L}[(b+\Delta b)\mathit{sin}\varphi +\frac{c}{2}+\frac{L}{\theta }]d\varphi =\frac{\pi \theta Ee{\left(b+\Delta b\right)}^{3}}{L}+\frac{2c\theta Ee{\left(b+\Delta b\right)}^{2}}{L}+4Ee{\left(b+\Delta b\right)}^{2}  \end{array}$$

Next we analyze the confinement layer, which is obtained by static balance:

25 $$\begin{array}{c}{F}_{3}={F}_{1}-{F}_{2}=P\left[\frac{1}{2}{\pi \left(b+\Delta b\right)}^{2}-\pi {m}^{2}\right]-{\int }_{0}^{\pi }\frac{2\theta Ee{\left(b+\Delta b\right)}^{2}\mathit{sin}\varphi }{L}d\varphi =P\left[\frac{1}{2}{\pi \left(b+\Delta b\right)}^{2}-\pi {m}^{2}\right]-\frac{4\theta Ee{\left(b+\Delta b\right)}^{2}}{L} \end{array}$$

Then the torque generated by the restraining layer is:

26 $$\begin{array}{c}{M}_{3}={F}_{3}{L}_{3}={F}_{3}\frac{L}{\theta }=\frac{L}{\theta }P\left[\frac{\pi }{2}{\left(b+\Delta b\right)}^{2}-\pi {m}^{2}\right]-4Ee{\left(b+\Delta b\right)}^{2} \end{array}$$

Combining (6), (13), (24), (26) $\theta$ can be expressed as

27 $$\begin{array}{c}\theta =\frac{L\left[\frac{2}{3}P{\left(b+\Delta b\right)}^{3}+\frac{1}{4}Pc\pi {\left(b+\Delta b\right)}^{2}-P\pi {m}^{2}\left(m+\frac{c}{2}\right)\right]}{\pi Ee{\left(b+\Delta b\right)}^{3}+2cEe{\left(b+\Delta b\right)}^{2}} \end{array}$$

Since each chamber is assumed to be uniformly deformed and the bending of SPA is continuous, the total bending angle of SPA can be obtained under the condition of inflation:

28 $$\begin{array}{c}\alpha =12\theta =f\left(P\right) \end{array}$$

**Figure 6:**
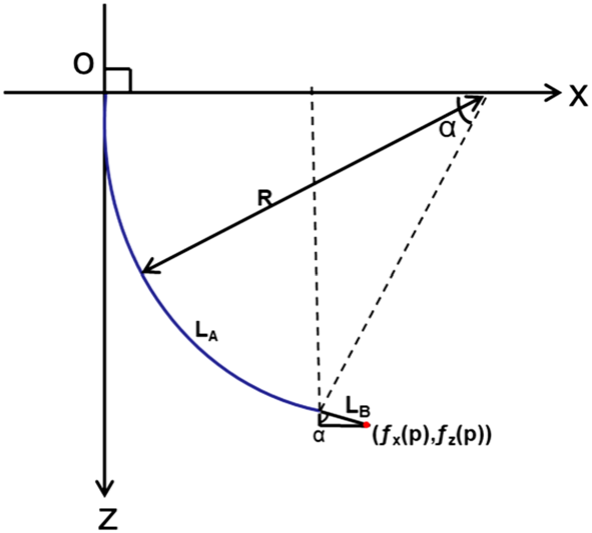
End position of a single SPA.

The position of the end of the SPA can be obtained according to the geometric relationship illustrated in Fig. 6.

${L}_{A}$ is the total length of the chamber, and ${L}_{B}$ is the tip length:

29 $$\begin{array}{c}{f}_{x}\left(P\right)=R-R\mathit{cos}\alpha =\frac{{L}_{A}}{\alpha }\left(1-\mathit{cos}\alpha \right)+{L}_{B}\mathit{sin}\alpha \end{array}$$

30 $$\begin{array}{c}{f}_{z}\left(P\right)=R\mathit{sin}\alpha =\frac{{L}_{A}}{\alpha }\mathit{sin}\alpha +{L}_{B}\mathit{cos}\alpha \end{array}$$

### Finite element analysis of SPA

Using a tensile test, the stress–strain relationship of the silica gel material is obtained. We compare the strain energy density function Yeoh three-parameter model with the best effect, ${C}_{10}=90036Pa$,${C}_{20}=-3880.6Pa$,${C}_{30}=1524Pa$, density $\rho =1072\mathrm{kg}/{\mathrm{m}}^{3}$.

**Figure 7:**
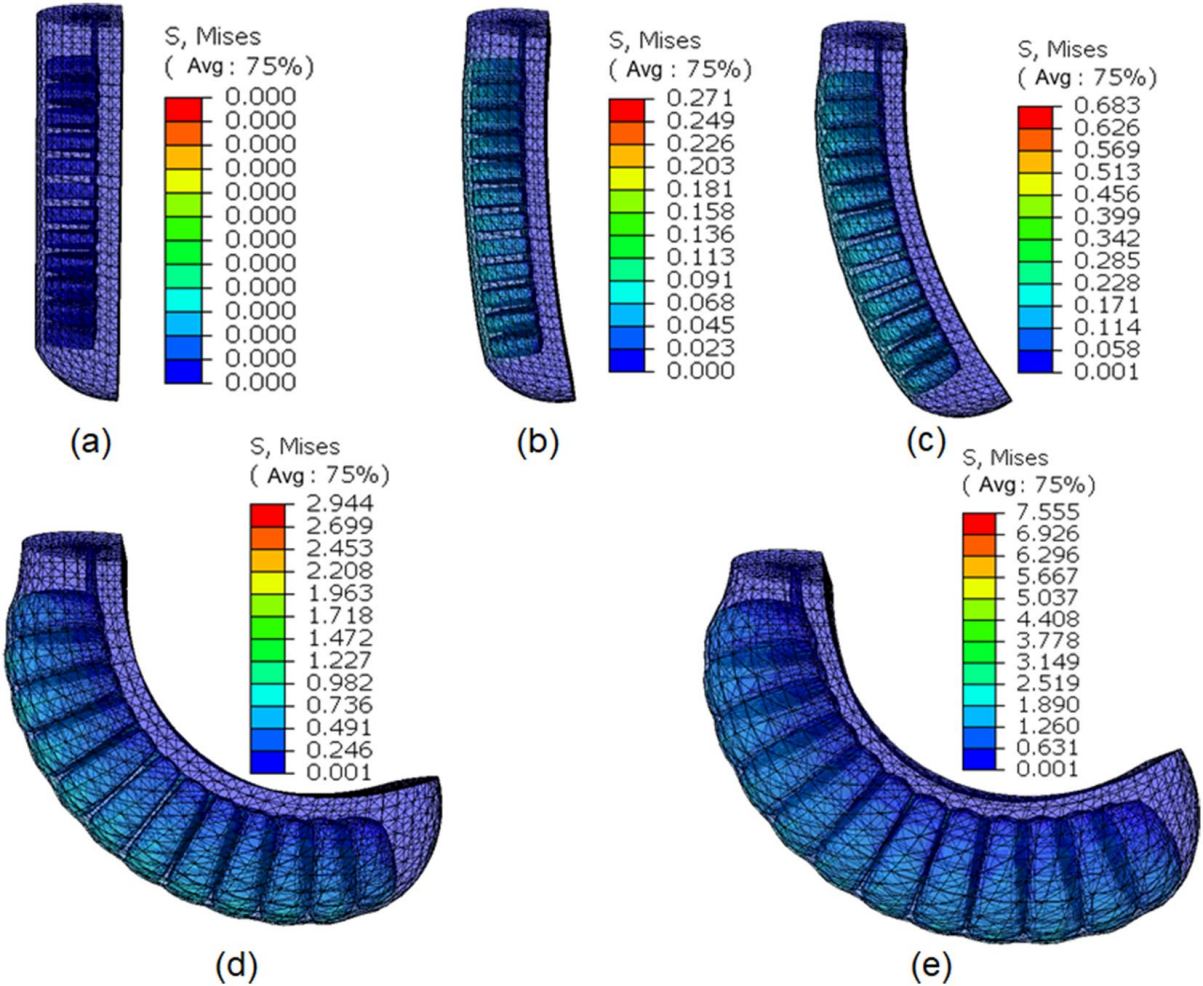
FEA of SPA. Based on the Abaqus software, the relationship between air pressure and curvature is simulated. From (**a**) to € is a simulation diagram with a pressure gradient from 0 to 100 kPa per 25 kPa.

In this paper, the finite element analysis (FEA) method is used to visually observe the SPA deformation and estimate its complete motion as a criterion for evaluation. We performed FEA using the software Abaqus to obtain the relationships between the deformation of the SPA, the end trajectory and the actuating pressure, as shown in Fig. 7.

### Experimental testing of SPA

**Figure 8:**
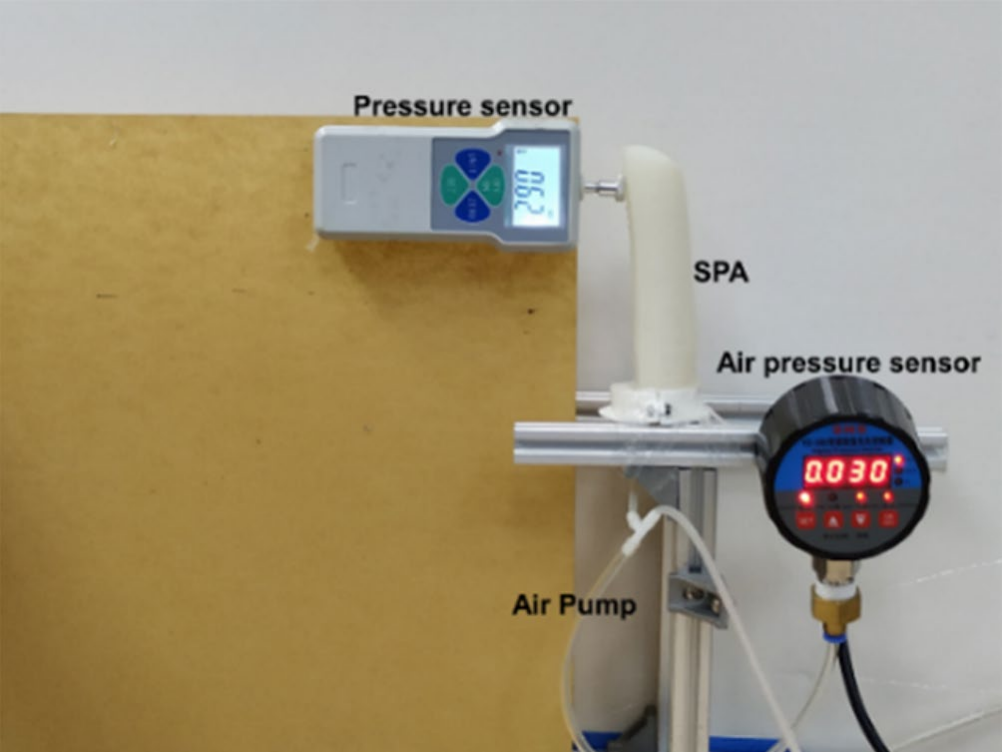
Experiments with SPA.

**Figure 9:**
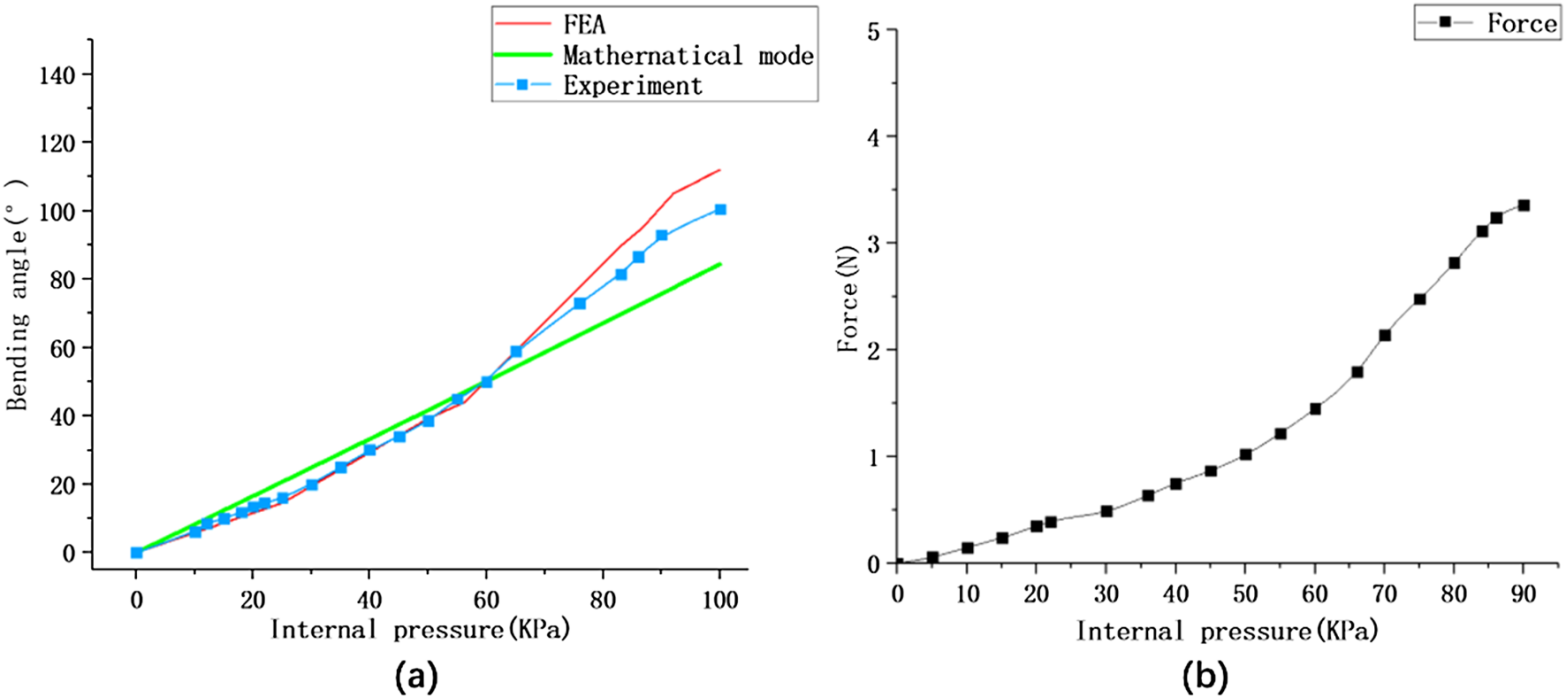
Experimental research on bending and end output force of SPA. (**a**) Comparison of the relationship between actuating pressure and curvature for simulations, mathematical models, and experiments. (**b**) Plot of actuation air pressure versus end force output for a single SPA.

As shown in Fig. 8, the end force test and bending test are carried out. In the experiment, the top of SPA is fixed on a frame, and the whole experiment process is photographed by a camera. The relationship between the end force of SPA and the air pressure can be obtained through the pressure sensor and air pressure sensor. After the pressure sensor is removed, the relationship between curvature and driving pressure can be obtained by processing the video. The results shown in Fig. 9.

The bending experimental results are basically consistent with the results of the mathematical model and finite element analysis before 60kpa, and there are differences after 60kpa. The reason is that some analysis is simplified in the mathematical model, the main reason is that the influence of thin wall thinning on resistance is not considered, and the elastic modulus is considered to be a constant. In the finite element analysis, it is because of the difference between the actual working condition and the ideal working condition, and some features are simplified, such as ignoring the fact that the SPA and the fixed parts may slide and extrude in the case of large bending under the actual working condition.

**Figure 10:**
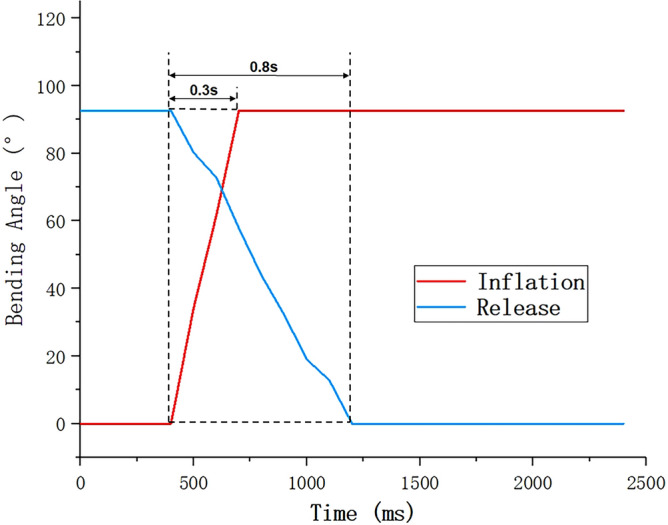
Step responses of SPA.

Step signal response is an important dynamic performance of SPA, which reflects the speed of SPA from sleep to work. In this paper, the motion capture system is used to capture the process of the SPA filling compressed air instantaneously. As shown in Fig. 10, it takes 0.3 s for the SPA to bend from 0° to 92.6° under the action of step signal, i.e. instantaneous filling of compressed gas. When the compressed gas is released, due to the non-linear material properties of the SPA, the mechanical fatigue effect will be produced. It takes 0.8 s to recover from 92.6° to 0°.

The experimental results show that the bending and output force of SPA are relatively stable, the bending performance meets the design requirements, and SPA has superior dynamic performance under the action of step signal, which can meet the requirements of fast grabbing.

### Grasping range and strategy of soft robot

**Figure 11:**
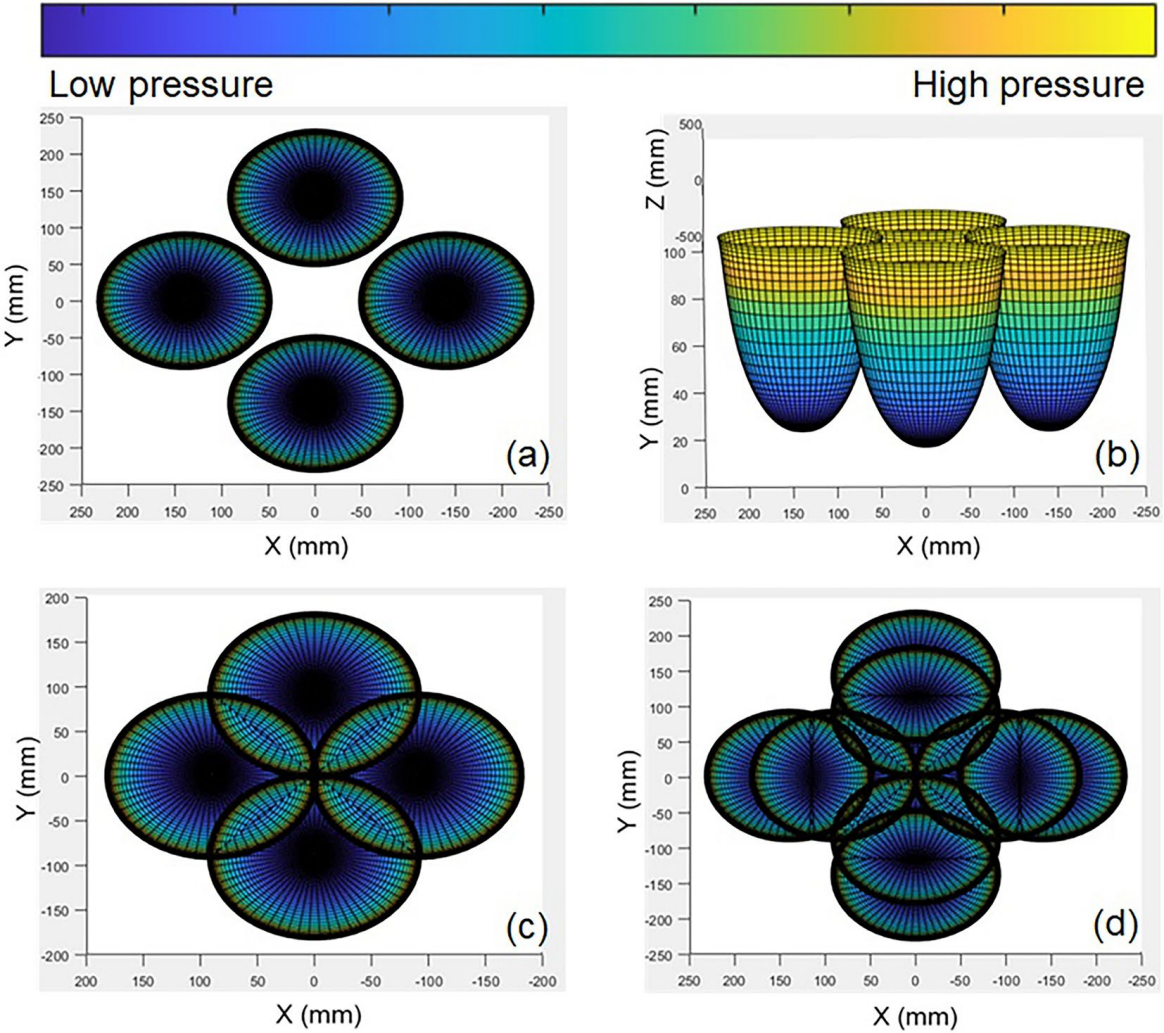
Three-dimensional workspace of the SPAs. (**a**) Workspace with the maximum spacing; (**b**) Stereogram for workspace; (**c**) Workspace with the smallest spacing; (**d**) Superposition graphs of maximal and minimal spaces.

According to minimum and maximum spacing of variable structure platform and SPA (Eqs. (29), (30), the minimum and maximum working space of 0–100 kPa end space position of SPA are shown in Fig. 11. The color indicates the magnitude of the actuating air pressure, where the closer to yellow the actuating air pressure is, the larger the pressure. At 100 kPa, the maximum grasping space is at least 300 mm in diameter and the minimum is 0 mm in diameter, which means that the SPA can reach most of the space. The variable structure greatly improves the space position that the end of SPA can reach. Visualization of the mathematical simulation software (MATLAB) shows the superiority of the grasping space and the grasping method of the variable-structured robot.

The motion space is not fixed. By increasing and changing the size of the $\Phi$ flange and the U-shaped crank, more variable structure space can be obtained. The grasp space model here is only used to validate the size of the design Grasping Strategy.

**Figure 12:**
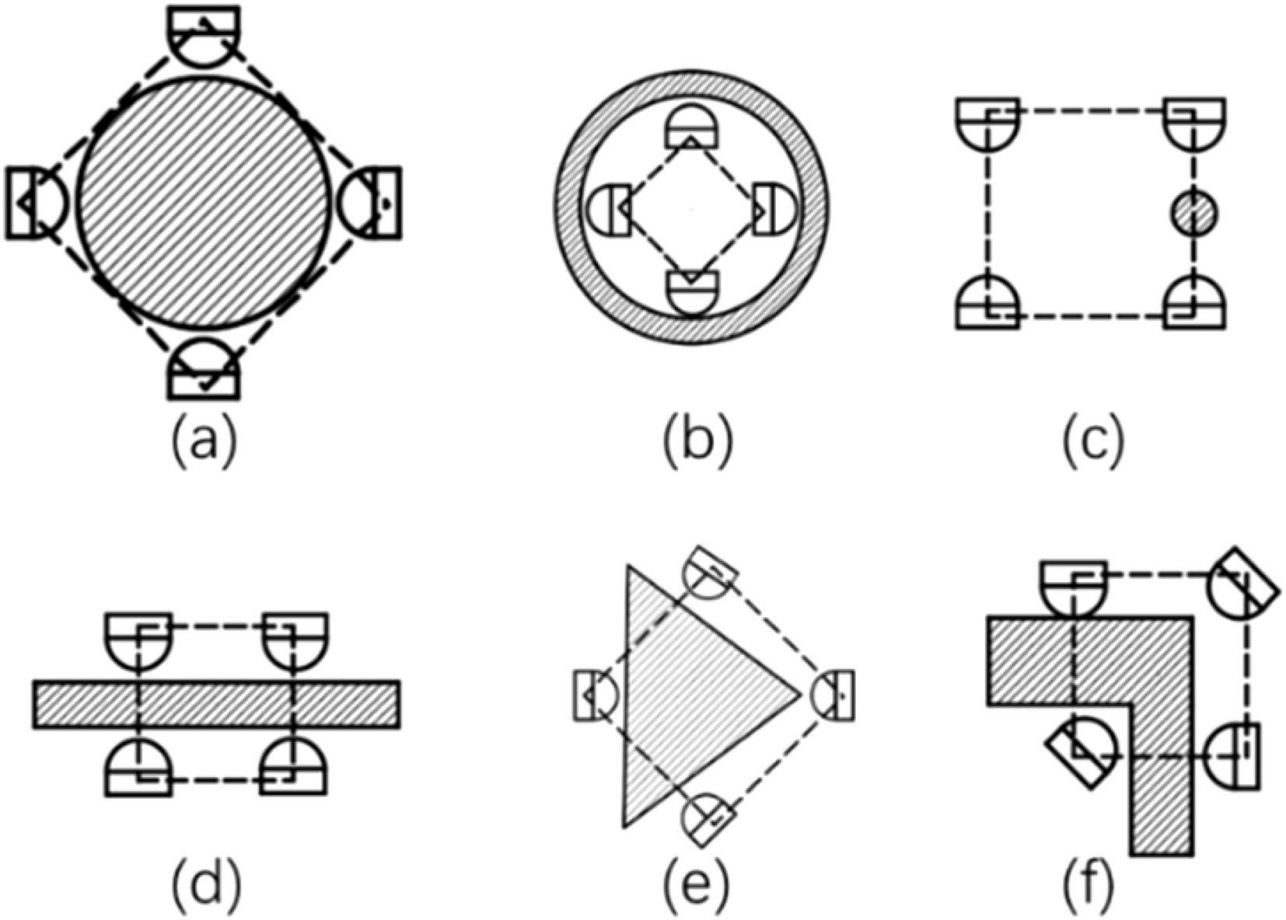
Schematic diagram of grasp strategy. (**a**) Circular object, (**b**) Ring object, **c** Smaller objects, (**d**) Long bar, (**e**,**d**) Other shape objects. The square dotted line represents the relative position information of different SPAs.

Since each finger can rotate independently, and the palm of SPA can be enlarged or reduced, the mechanism has different grasping strategies according to the shape and size of the given object. Several examples are given in Fig. 12.

The gripper can provide a variety of gripping strategies. As shown in Fig. 12a–d, different grasping modes correspond to objects with different characteristics, such as four SPAs centering modes, which are mainly used for objects with uniform aspect ratio of circular, spherical and cubic shapes; Under the reverse expansion mode, for large objects with groove or ring features, centering grasps are used for ring objects; The double finger gripper can effectively improve centering ability as hands. The gripper is easier to grasp small or light objects, such as hexagon socket, card, paper, etc.; Parallel mode can grasp objects with large aspect ratio through other modes, such as cuboid box, long circular tube, etc., compared with other modes, it can grasp more stably.

**Figure 13:**
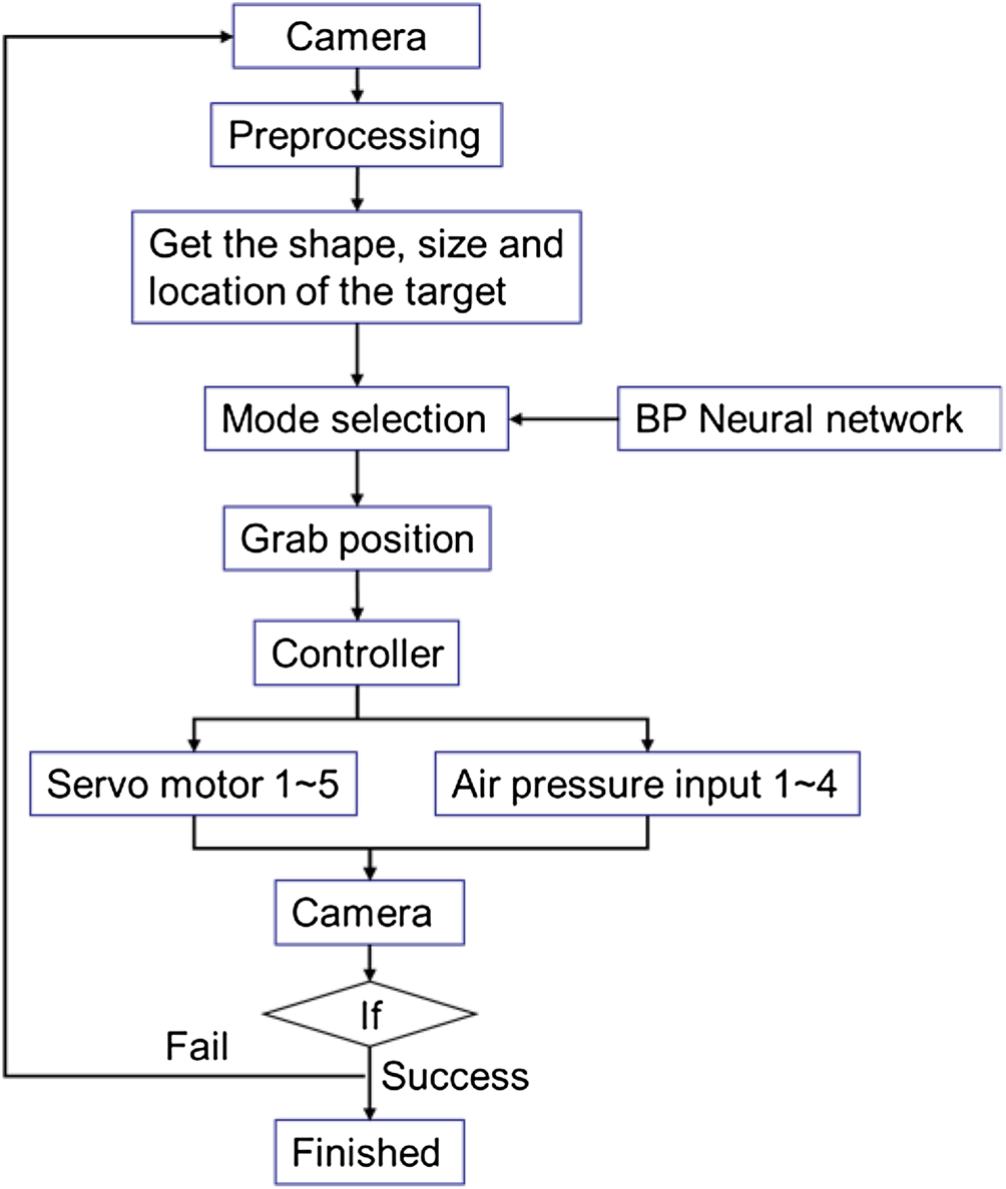
Fetching strategies.

In order to capture the target, this paper proposes a simple strategy network as shown in Fig. 13, which is composed of image recognizer and control strategy. Through image preprocessing, the image is filtered, denoised and binarized, and the characteristic parameters of the image are obtained. The grasping mode is obtained by the image recognizer, and the corresponding grasping position is obtained. Input the output parameters into the controller, respectively control the 1 ~ 5 servo motor and air pressure values of fingers 1 ~ 4, then judge the success through the camera. If not, return to the first step.

**Figure 14:**
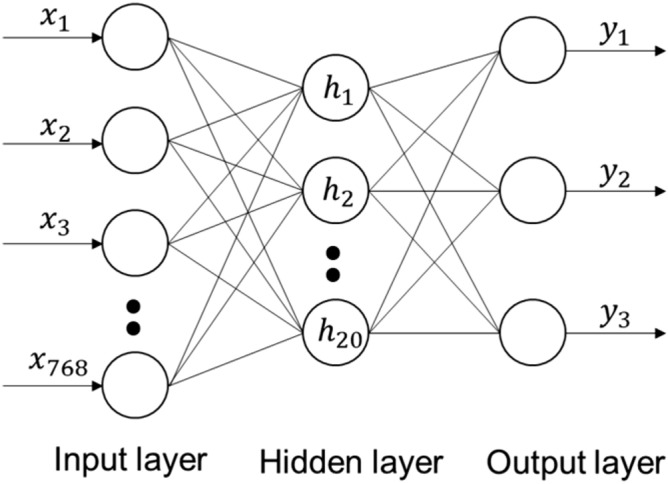
The simplified BP neural network diagram.

As shown in the Fig. 14, the recognizer is a three-layer back propagation(BP) neural network, which is a feed-forward network trained by error back propagation algorithm and output through sigmoid excitation function.

Sigmoid function:

31 $$\begin{array}{c}\sigma \left(a\right)=\frac{1}{1+{e}^{-a}} \end{array}$$

The output of each unit in the middle layer is:

32 $$\begin{array}{c}{h}_{j}=\sigma \left(\sum_{i=0}^{N-1}{V}_{ij}{x}_{i}+{\phi }_{j}\right) \end{array}$$

The output of each unit in the output layer is:

33 $$\begin{array}{c}{y}_{k}=\sigma \left(\sum_{i=0}^{L-1}{W}_{jk}{h}_{j}+{\theta }_{k}\right)\end{array}$$

where the number of input layer units N = 768, number of middle layer units L = 20, unit number of output layer M = 3. Input vector $\mathrm{X}=({\mathrm{x}}_{1},{\mathrm{x}}_{2},\dots ,{\mathrm{x}}_{748})$ is the feature of the object being caught, H $=({\mathrm{h}}_{1},{\mathrm{h}}_{2},\dots ,{\mathrm{h}}_{20})$ is the output vector of the middle laye, output vector Y $=({\mathrm{y}}_{1},{\mathrm{y}}_{2},{\mathrm{y}}_{3})$ is the gripping mode of the gripper, which is divided into two fingers mode, general mode and backhand mode. The weight from input unit i to hidden unit j is ${V}_{ij}$, the weight from hidden unit j to output unit k is ${W}_{ij}$, and ${\uptheta }_{k}$ and ${\upphi }_{j}$ are the thresholds of output unit and hidden unit respectively.

**Figure 15:**
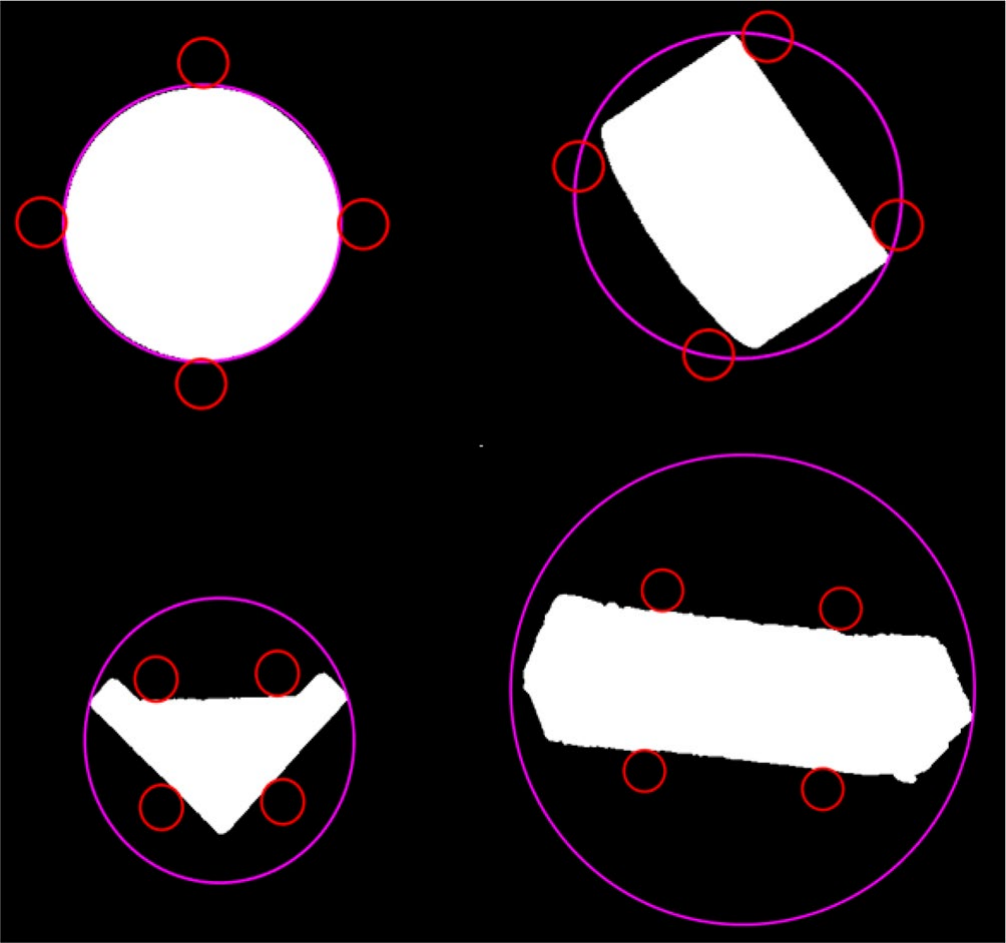
Recognition of grasp position for four objects with different features.

As shown in the Fig. 15, the image processing is based on MATLAB software, taking the common mode as an example for the grasp position recognition, a minimum square is made in the geometric center of the feature parameter, and the four vertices of the square mark the circle of the same size of SPA. By continuously rotating and scaling the square, the four circles are tangent to the grasped object, and the square cut length and the tangent point position of the four circles and the grasped object are obtained. The final output is clamping The SPAs spacing of the device and the rotation angle of the four SPAs.

### Experimental verification

**Figure 16:**
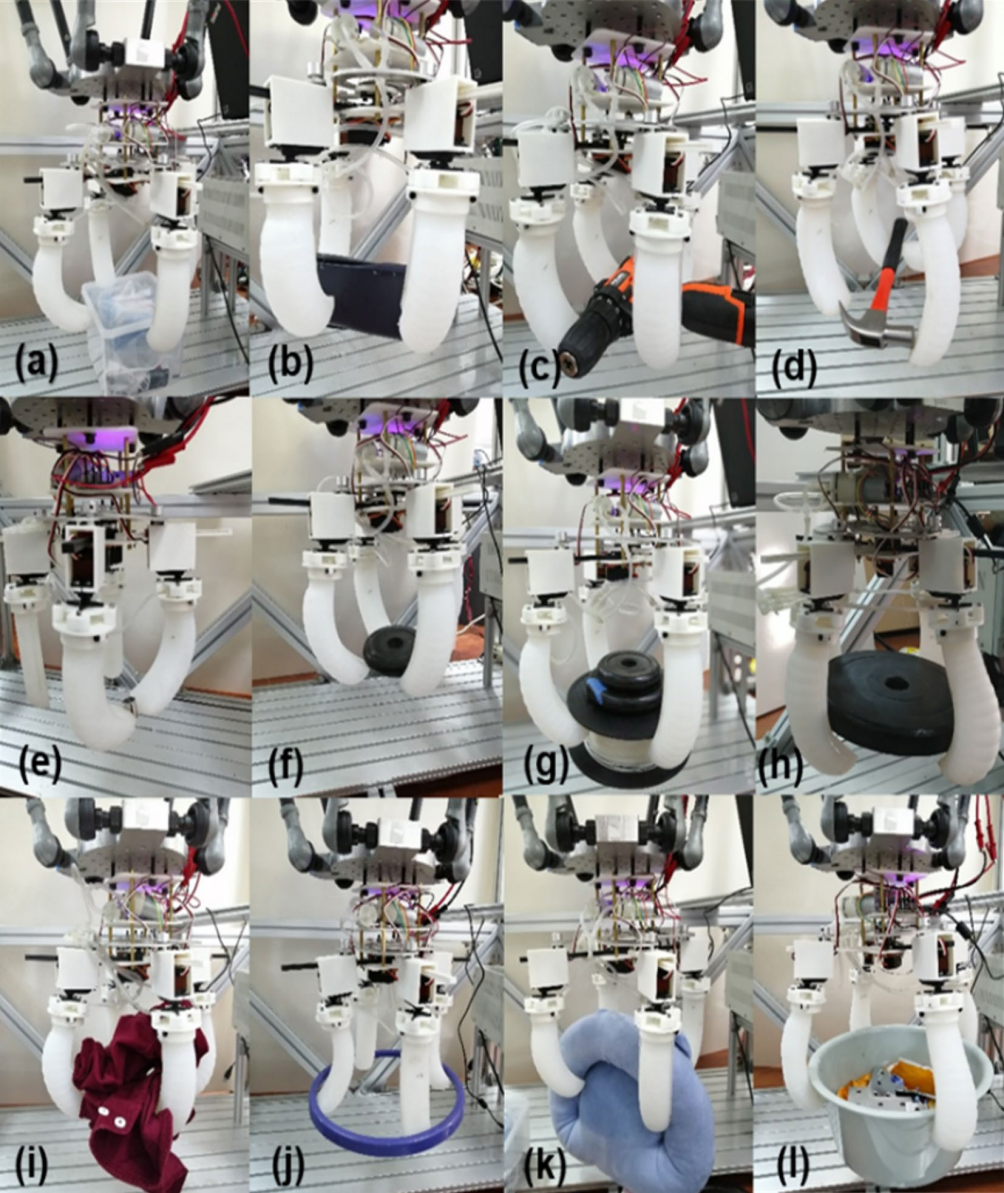
Experiments with different objects in multiple modes. (**a**) Box; (**b**) calculator; (**c**) electric drill; (**d**) hammer; (**e**) hexagon; (**f**) small clump weight; (**g**) 3D printing consumables; (**h**) large clump weight; (**i**) article of clothing; (**j**) ring; (**k**) pillow; (**l**) pot.

Some representative grasping work was summarized as shown in Fig. 16 and the corresponding parameters of the captured object are shown in Table 2. The experiment was repeated 10 times with each object, all of which showed it was effective for grasping, except that the success rate of the h grasp experiment was only 30%, (Generally, grasping the object for ten seconds without dropping is considered an effective grasp [33]). Other grasps could grasp for a long time without falling off, and the success rate of grasping was 100%. The main problem was with the clump weight in experiment h. This clump weight is smooth, and the gripper’s friction is not enough to support the load of 25 N. If the actuating air pressure is increased, the load capacity of the gripper will be increased, although this will reduce the service life of the gripper. The main method is still to increase the surface friction of the holder and to use a material with a higher elastic modulus. The experiment part can be seen in the Supplementary Video (Experimental video), including response experiment, correlation experiment between end force and air pressure, and grabbing experiment.

**Table 2:** Experimental object of gripper grasps.

Number	Target		Size (mm)	Weight (g)
a	Box		150 × 150 × H52	552
b	Calculator		165 × 190 × H85	230
c	Electric drill		191 × 191 × 50	961
d	Hammer		230 × 100 × H30	331
e	Hexagonal sleeve		Φ8 × H10	10
f	Small clump weight		Φ105 × H22	350
g	Consumables		Φ133 × H22	660
h	Large clump weight		Φ208 × H25	2500
i	Clothes		Arbitrary shape	200
j	Ring		Φ346 Φ270	30
k	Pillow		350 × 250	300
l	Pot		Φ310 × 130	2300

Grasping experiments with different diagonal distances are represented by different colors. The object to be grasped is a number of disc flakes of different diameters. The abscissa represents the diameter of the object, and the ordinarily represents the weight of the object. The green area represents the gripping area of the gripper.

**Figure 17:**
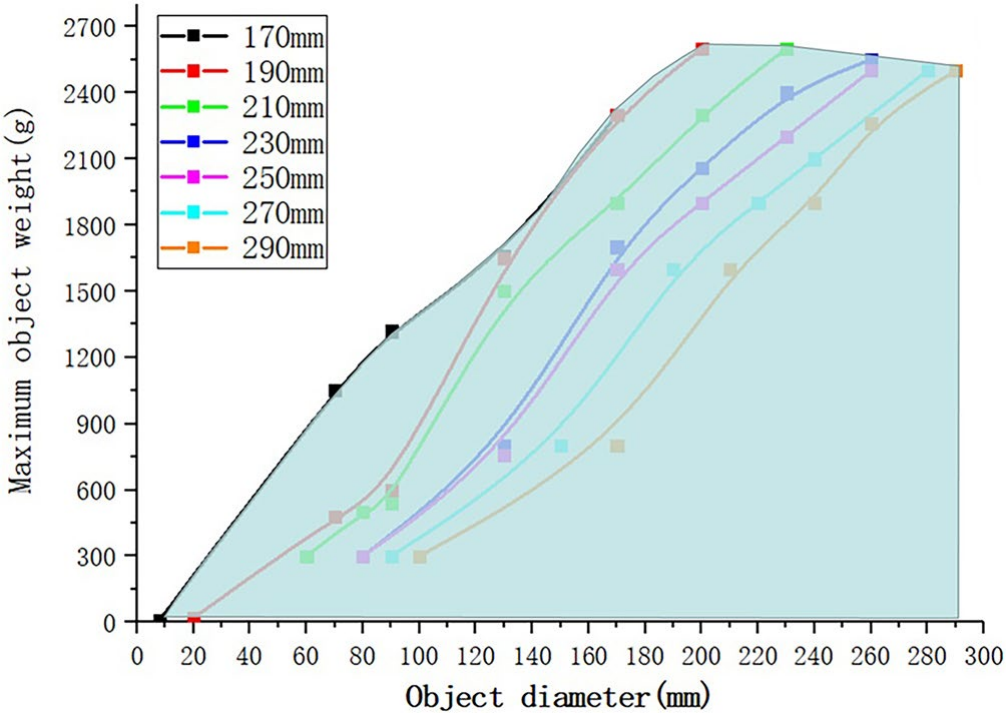
Comparative experiment of grasping ability under different distances between SPAs.

In order to verify that the expansion and contraction ability of the gripper can effectively adapt to different object sizes, we have done a series of comparative experiments as shown in Fig. 17. In the four fingers mode, we place heavy objects on the disk. When the gripper fails to hold, the heavy objects fall. By weighing the limit grasping weight of the gripper, the final grasping weight of the gripper for different diameter objects under different spacing is obtained, and the data are fitted linearly. The experimental results showed that with the increase of SPA spacing, the diameter and weight of the object that can be grasped are also increasing. The ultimate grasping weight is about 2500 g when the distance is greater than 190 mm, which is due to the limited load of SPA. There are some differences between the experimental results and the actual gripping ability, which is caused by the deviation of the gripping position and the uneven placement of the heavy objects, but it still shows that the expansion and contraction ability of the gripping device can effectively adapt to different object sizes. Compared with the fixed spacing gripping device, it can effectively improve the ultimate gripping weight and range of the gripper.

Generally, through the joint action of two kinds of variable structures, the grasping strategy and space of the gripper is effectively improved. If there is no variable structure, the gripper can only passively grasp objects adapted to a fixed diagonal distance, but now it can actively adapt to 8–310 mm irregular objects. The weight of the gripper has also made great progress, and has a high adaptability and success rate.

## Conclusion and future works

In this paper, an adaptive soft clamping mechanism with variable structure is proposed. The mechanism optimizes the clamping strategy in the way of active adaptation of variable structure to solve the shortcomings of the current soft clamping strategy. The design and manufacturing method of the soft actuator have been introduced. The mathematical model of the single SPA and the kinematics model of the clamping mechanism are deduced. Experiments measuring bending and end force were carried out to verify the validity of the model, grasping test and step response experiment of SPA was carried out. The experimental results prove that the soft robot we designed can clamp a weight of at most 2.5 kg and objects of 8 mm to 310 mm in diameter. The SPA spacing can be expanded from 200 to 300 mm, and the flexibility of the mechanism is verified by grasping various objects, in particular demonstrating its powerful grasping strategy and grasping range. Although the soft robot is only a pre-production prototype, several advantages such as safety and adaptability have already emerged. If it is optimized to obtain higher performance, the soft gripper will be suitable for real-life applications. Being able to grip a variety of objects has high application value and provides a guiding idea for the development of future soft grippers.

Plans for future work include further improvement of the soft gripper design and improved stability of the mechanism through synovial control.

## Supplementary information

Supplementary Video 1.
